# Novel sirolimus-eluting stent Prolim® with a biodegradable polymer in the all-comers population: one year clinical results with quantitative coronary angiography and optical coherence tomography analysis

**DOI:** 10.1186/s12872-015-0139-5

**Published:** 2015-11-14

**Authors:** Jacek Bil, Robert J. Gil, Adam Kern, Tomasz Pawłowski, Piotr Seweryniak, Zbigniew Śliwiński

**Affiliations:** Department of Invasive Cardiology, Central Clinical Hospital of the Ministry of Interior, 137 Woloska Street, 02-507, Warsaw, Poland; Institute of Experimental and Clinical Medicine, Polish Academy of Science, Warsaw, Poland; Faculty of Medical Sciences University of Varmia and Masuria, Olsztyn, Poland

**Keywords:** Sirolimus-eluting stent, Biodegradable polymer, OCT, Prolim® stent

## Abstract

**Background:**

The aim of this study was to assess the safety and the efficacy of the novel sirolimus-eluting Prolim® stent with a biodegradable polymer in the all-comers population.

**Methods:**

We prospectively enrolled all patients with stable coronary artery disease or acute coronary syndrome treated with Prolim® stent between January and December 2013 in two interventional cardiology centers in Poland. Angiographic control was planned at 12 months, in which 15 % of patients (randomly chosen) underwent optical coherence tomography imaging. The primary end-point was the cumulative rate of cardiac death, myocardial infarction, and target lesion revascularization at 12 months.

**Results:**

There were 204 patients enrolled, in whom 238 Prolim® stents were deployed (1.17 stent per patient). The mean age was 68 ± 10 years and 32.8 % were females. The examined stent was implanted in 5.9 % in STEMI patients, in 21.6 % - in NSTE-ACS and in 72.5 % - in patients with stable coronary artery disease. The Prolim® stent was most frequently implanted in right coronary artery (38.2 %) followed by left anterior descending artery (34.0 %). The cumulative major adverse cardiovascular events rate at 12 months was 6.9 %, and the clinically-driven target lesion revascularization rate – 5.4 %. At 12 months in quantitative coronary angiography the late lumen loss was 0.21 ± 0.18 mm, and in optical coherence tomography the mean neointima burden was 24.6 ± 8.6 %.

**Conclusions:**

Sirolimus-eluting Prolim® stent with a biodegradable polymer is a feasible device with a very good safety profile and long-term clinical effectiveness.

**Trial registration number:**

ClinicalTrials.gov NCT02545985.

## Background

Drug eluting stents (DES) reduce the incidence of restenosis and thereby also the incidence of repeated revascularizations. Simultaneously, DES also impair proper healing and endothelialization, what might lead to the increased risk of late and very late stent thrombosis [1]. In 2003 Polish physicians and engineers created the concept of covering bare metal stents with a biodegradable polymer [2]. This was associated with the discovery of the negative impact of permanent polymers of the first generation DES on the vessel wall, which resulted in impaired healing and endothelialization [3]. The idea assumed that the biodegradable polymer would be absorbed from the stent surface after drug elution, and the remaining bare metal stent platform covered with neointima would not evoke further irritation to the artery wall [4]. Preclinical observations in the porcine model proved favorable vessel healing after Prolim® stent deployment [5].

The aim of this study was to assess the safety and the efficacy of Prolim® stent in the all-comers population.

## Methods

### Study population and study design

It was a prospective, single-arm, open-label clinical study, in which patients were enrolled in two invasive cardiology centers in Poland (Warsaw and Olsztyn) between January and December 2013. The blinded data were entered into the electronic case report form by collaborating physicians in these centers. The inclusion criteria were: age ≥ 18 years old, stable coronary artery disease (SCAD) or acute coronary syndrome (unstable angina – UA, non-ST elevation myocardial infarction – NSTEMI or ST-elevation myocardial infarction – STEMI) and the signed informed consent. Main exclusion criteria were: inability to take dual antiplatelet therapy for 12 months, left ventricular ejection fraction ≤ 30 %, chronic total occlusions, and in-stent restenosis. The Ethics Committee of the Central Clinical Hospital of the Ministry of Interior in Warsaw approved the study protocol (ClinicalTrials.gov Identifier: NCT02545985).

### Study device

**Fig. 1:**
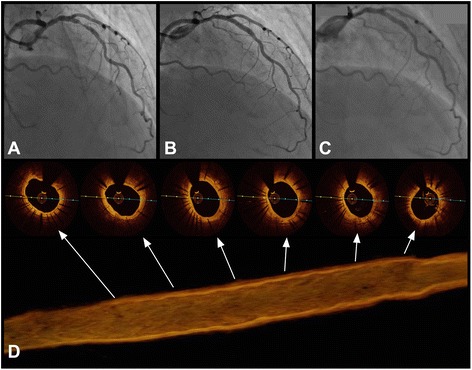
The Prolim® stent. **a** Lesion in LAD before stent implantation, **b** LAD directly after Prolim® 3.5 × 15 mm deployment, **c** LAD at 12 months after stent implantation, **d** OCT analysis of Prolim® stent at 12 months

The Prolim® stent is a balloon expandable coronary stent with RX delivery system. The stent platform is made of a laser-cut 316 L metallic tube with a wall thickness of 115 μm. This stent is characterized by a relatively high radial force (8.5 – 9 PSI). The stent diameter ranges from 2.0 mm to 5.0 mm, and length - from 8 mm to 40 mm. The profile of the whole implantation system, including stent, is 0.038". The nominal shortening of the stent during stent expansion is low (<0.5 %). The Prolim® stent is covered with a structure containing a co-polymer of lactic and glycolic acids mixed with a solvent and the drug - sirolimus (1.2 μg/mm^2^). The coating degrades almost entirely within 8 weeks [5] [Fig. 1].

### Procedure

Percutaneous coronary interventions (PCI) were performed according to local standards via radial or femoral access using 6 Fr or 7 Fr guiding catheters. Pharmacological treatment was according to the most recent guidelines. Troponin I (TnI), creatine kinase (CK) and creatine kinase-myocardial band (CK-MB) were measured pre-procedural and after 6 h and 24 h postprocedure in all patients. Periprocedural myocardial infarction (type 4a) was defined according to the third universal definition [6].

### Follow-up

The assessment of the anginal status, data collection of adverse events, details of any subsequent coronary interventions, and the use and changes in concomitant medications were collected at 30 ± 7 days and 12 ± 0.5 months. The angiographic control was planned at 12 months, in which 15 % of patients (randomly chosen) underwent optical coherence tomography (OCT) examination.

### Endpoints

The primary endpoint was the cumulative rate of major adverse cardiovascular events (MACE) consisting of cardiac death, myocardial infarction (MI) and clinically-driven target lesion revascularization (TLR). Secondary endpoints included cardiac death, all-cause death, MI, TLR, target vessel revascularization (TVR), stent thrombosis, late lumen loss (LLL) assessed in quantitative coronary angiography (QCA), the percentage of covered struts and neointima volume and morphology characteristics assessed in OCT, as well as the device success rate. Cardiac death included death resulting from an acute MI, sudden cardiac death, death due to heart failure and death due to cardiac procedures. All deaths were deemed cardiac unless proven otherwise. MI was defined according to third universal definition [6]. Clinically-driven TLR was defined as reintervention of the target lesion due to presence of a symptomatic ≥ 50 % diameter stenosis during follow-up. TVR was defined as any revascularization of any segment of the index coronary artery. Device success was defined as successful deployment of the intended stent in the target site without a system failure. The definite stent thrombosis was defined as state with symptoms suggestive of an acute coronary syndrome and angiographic or pathologic confirmation of stent thrombosis. The probable stent thrombsis was defined as the unexplained death within 30 days or target vessel myocardial infarction without angiographic confirmation of stent thrombosis, and the possible stent thrombosis was defined as any unexplained death after 30 days [7].

### Quantitative angiography analysis

All coronary angiograms were recorded after an intracoronary administration of 200 μg of nitroglycerin. Two orthogonal views were chosen to visualize the target lesion. A QCA analysis was performed using commercially available software (QCA-CMS version 5.0, Medis, Leiden, the Netherlands). Catheter calibration was used in all cases. The following parameters: lesion length, reference vessel diameter, minimal lumen diameter, % diameter stenosis, acute lumen gain and LLL were calculated as described previously [8]. The analysis was performed independently by two interventional cardiologists: TP and JB.

### Optical coherent tomography analysis

Time-domain OCT examinations were performed using a well validated non-occlusive technique [9]. Briefly, after wiring the artery with the guidewire as described previously, the Dragon Fly catheter (LigthLab Co.) was advanced distally to the implanted stent and during continuous contrast media flush (Iodixanol, Visipaque GE Healthcare), the automatic pullback was performed. The commercially available console (M2 or M3 by LigthLab Co.) was used.

Optical coherence tomography images were obtained along the region of interest, which was the implanted stent plus 5 mm both proximal and distal. Off-line analysis was performed after careful recalibration of acquired images along the reconstructed longitudinal segment. Calibration was obtained by adjusting the z-offset, the zero-point setting of the system. The analysis was performed applying a dedicated off-line software (St Jude Medical). Quantitative measurements of the minimal lumen area and minimal lumen diameter were obtained in all consecutive frames along the region of interest using semi-automated algorithm. Additionally, the mean value of all lumen area cross-sections measured inside the region was calculated. Additionally, lumen volume analysis was performed along region of interest - all measured lumen area cross-sections were summed. Mean neointimal burden was calculated as the ratio of the mean neointima area to the mean stent area.

Moreover, to assess stent apposition OCT analysis was performed every 0.2 mm of the stent. The stent struts apposition was classified as: 1) apposed 2) protruded and 3) malapposed according to a distance length between vessel wall and center of the stent strut. If such distance was: 1) more than 130 μm, malapposition was detected, 2) in range of 20 to 130 μm, protrusion was detected. The morphology of the neointima was analyzed according to the previously validated OCT criteria, and classified as type I (thin cap neoatheroma, lipid-rich), type II (thick-cap, layered), type III (peri-strut, homogenous) and type IV (pre-existing, homogenous) [10, 11]. The analysis was performed independently by two interventional cardiologists: TP and JB.

### Statistical analysis

Continuous variables were presented as mean ± standard deviation. Categorical data were presented as numbers (%). Continuous variables were compared using an unpaired student two-sided t test, and categorical data using the χ^2^ test or Fisher exact test, as appropriate. If distribution was not normal (verified with the Shapiro–Wilk test), Wilcoxon signed-rank tests and Mann–Whitney U-tests were used. P values of < 0.05 were considered statistically significant. Statistical analyses were performed using R 3.0.2 for OS (R Foundation, Vienna, Austria).

## Results

### Baseline clinical and angiographic characteristics

A total of 204 patients were enrolled into this study. The mean age was 68 ± 10 years, and 28.8 % of patients were women (*n* = 67). The reasons for PCI were: symptomatic SCAD (72.5 %, *n* = 148) followed by NSTEMI (11.8 %, *n* = 24), unstable angina (9.8 %, *n* = 20) and STEMI (5.9 %, *n* = 12). The detailed clinical characteristic is presented in Table 1.

**Table 1:** Baseline clinical characteristics

Baseline clinical characteristics	*n* = 204 (%)
Age [years]	68 ± 10
Women	67 (32.8)
Hypertension	177 (86.7)
Hypercholesterolemia	174 (85.1)
Diabetes type 2	76 (37.3)
Prior MI	60 (29.4)
Prior PCI	93 (45.6)
CABG	24 (11.8)
Chronic kidney disease	27 (13.2)
Clinical indication for PCI	
planned PCI	148 (72.5)
UA	20 (9.8)
NSTEMI	24 (11.8)
STEMI	12 (5.9)

*MI* myocardial infarction, *PCI* percutaneous coronary intervention, *CABG* coronary artery bypass graft, *UA* unstable angina, *NSTEMI* non-ST-elevation myocardial infarction, *STEMI* ST-elevation myocardial infarction

In most cases patients presented with the multivessel disease (62.3 %) and lesions of the moderate complexity (type A – 31.4 %, type B1 – 31.9 %). Lesions were located most frequently in right coronary artery (38.2 %, *n* = 78) and in the left anterior descending artery (33.8 %, *n* = 69). In 38.8 % (*n* = 79) of cases lesions within coronary bifurcation were treated. More details are presented in Table 2.

**Table 2:** Baseline angiographic characteristics

Parameter	*n* = 204 (%)
Multivessel disease	127 (62.3)
Functional LIMA on LAD	22 (10.8)
Lesion type	
A	64 (31.4)
B1	65 (31.9)
B2	39 (19.1)
C	36 (17.6)
Lesion location	
LM	14 (6.9)
LAD	69 (33.8)
LCx	35 (17.2)
RCA	78 (38.2)
VG	8 (3.9)
Bifurcation lesions	
Side branch > 2 mm	45 (22.1)
Side branch < 2 mm	34 (16.7)
none	125 (61.2)
Vessel tortuosity	
None – mild	123 (60.3)
Moderate – severe	81 (39.7)
Calcification	
None – mild	143 (70.1)
Moderate - severe	61 (29.9)

*LIMA* left internal mammary artery, *LAD* left anterior descending artery, *LM* left main stem, *LCx* left circumflex artery, *RCA* right coronary artery, *VG* venous graft

### Procedural characteristics

The main procedural variables are presented in Table 3. The device success rate was 99 %. There were two delivery failures in which the stent was deformed due to heavy calcification, but did not fall from the balloon. After safe removal of the stent delivery system and further predilatations patients were successfully treated with the new Prolim® stent. The mean nominal Prolim® stent parameters were: 3.22 ± 0.52 mm and 18.41 ± 5.41 mm, while the mean maximal implantation pressure was 15.2 ± 2.5 atm. On average 1.17 Prolim® stents were implanted per patient. The predilatation rate was 59.8 %, while the postdilatation rate was 27.5 % (mean pressure 20.1 ± 3.7 atm). Most postdilatations were performed with non-compliant balloons (49/56, 87.5 %). Almost all procedures were performed via the 6 F guiding catheter (99 %) and in 92.6 % - the radial access was preferred.

**Table 3:** Procedural characteristics

Parameter	*n* = 204 (%)
Device success	202 (99.0)
No of stents per patient	1.17
Predilatation	122 (59.8)
Postdilatation	56 (27.5)
Nominal stent diameter [mm]	3.22 ± 0.52
Nominal stent length [mm]	18.41 ± 5.41
Stent maximal inflation pressure [atm]	15.23 ± 2.54
Balloon to artery ratio	1.10 ± 0.07
Additional stent implantation due to dissection	14 (6.8)
Additional stent implantation due to lesion length	16 (7.8)
Vascular access radial/femoral	189 (92.6)/15 (7.4)
Guiding catheter 6 F/7 F	202 (99.0)/2 (1.0)

There were 34 bifurcation cases (16.7 %) with the side branch diameter < 2 mm. These cases were treated with provisonal T-stenting technique without protection of the side branch in 1,1,0 Medina classification. Additionally, there were 45 cases (22.1 %) of bifurcation with the side branch > 2 mm. In those patients provisonal T-stenting technique was used, however in 8 of them the procedure ended up with the second stent delpoyed in side branch using the T and protrude technique (TAP).

### Clinical outcomes

There was seven (3.4 %) periprocedural MI due to distal dissection and debris embolization. Additionally, there were 9 (4.4 %) patients with in-hospital increased TnI levels (max 1.6 ng/mL). These were all asymptomatic, without ECG changes and did not require to repeat coronary angiography (i.e. they did not meet the criteria of MI type 4a).

The clinical follow-up at 12 months was available in all patients (Table 4). The cumulative incidence of MACE was 6.9 % (*n* = 14). In the observation period there was no death or stent thrombosis. The MI rate was 1.5 % (*n* = 3). In detail, there were three MI cases after in-hospital period caused by one case of TLR and two cases were caused by new lesions in other coronary arteries. The clinically-driven TLR rate was 5.4 % (*n* = 11). All cases were treated by PCI (plain old balloon angioplasty – 4 cases, DES – 7 cases).

**Table 4:** Clinical results

Endpoints	30 days	12 Mo
	*n* = 204 (%)	*n* = 204 (%)
MACE	7 (3.4)^a^	14 (6.9)
Death	0	0
cardiac death	0	0
MI	7 (3.4)^a^	3 (1.5)
Stroke	0	1 (0.5)
ST	0	0
TLR	0	11 (5.4)
TVR	0	15 (7.4)
PCI in another vessel	8 (3.9)	19 (9.3)

*MACE* major adverse cardiovascular event, *MI* myocardial infarction, *PCI* percutaneous coronary intervention, *ST* stent thrombosis, *TLR* target lesion revascularization, *TVR* target vessel revascularization

^a^periprocedural MI type 4a

### Quantitative coronary angiography and optical coherence tomography analysis

The QCA data are presented in Table 5. The immediate angiographic success rate was 100 %. Acute lumen gain was 1.87 ± 0.41 mm. The 12-month follow-up angiography was available in 89 patients (43.6 %). Late lumen loss was 0.21 ± 0.18 mm.

**Table 5:** Quantitative coronary angiographic analysis

Parameter	Pre stenting	Post stenting	FU
lesion length [mm]	15.47 ± 1.94		
RVD [mm]	3.39 ± 0.24	3.41 ± 0.23	3.45 ± 0.25
MV - % DS	64.6 ± 15.3 %	9.6 ± 4.1 %*	16.8 ± 6 %**
MLD [mm]	1.21 ± 0.34	3.08 ± 0.28*	2.87 ± 0.37**
ALG [mm]		1.87 ± 0.41	
LLL [mm]			0.21 ± 0.18

QCA analysis based on *n* = 89 cases (43.6 %)

*RVD* reference vessel diameter, *% DS* % diameter stenosis, *MLD* minimal lumen diameter, *ALG* acute lumen gain, *LLL* late lumen loss, *FU* follow-up. * P < 0.05 between pre stenting and post stenting; ** P < 0.05 between post stenting and FU

The OCT analysis data are presented in Table 6. The OCT at 12 months was performed in 29 patients (14.2 %). After that period only 0.07 % of struts were uncovered and 0.1 % were malapposed. The nominal analyzed stent diameter was 3.25 ± 0.42 mm, and in OCT the stent diameter was 3.23 ± 0.38 mm. The mean neointima burden was 24.6 ± 8.6 % and the neointima volume was 28.16 ± 15.10 mm^3^.

**Table 6:** Optical coherence tomography analysis at 12 months

Stent apposition	10 484 struts (%)
Embedded	10342 (98.6)
Protruding	125 (1.2)
Uncovered	7 (0.07)
Malapposed	10 (0.1)
OCT parameters	
Nominal stent diameter [mm]	3.25 ± 0.42
Nominal stent length [mm]	13.67 ± 2.88
Mean minimal lumen area [mm^2^]	4.82 ± 1.41
Mean lumen area [mm^2^]	6.21 ± 1.10
Mean lumen diameter [mm]	2.79 ± 0.24
Mean stent area [mm^2^]	8.39 ± 2.26
Mean stent diameter [mm]	3.23 ± 0.38
Mean neointima area [mm^2^]	2.17 ± 1.37
Mean neointima thickness [mm]	0.19 ± 0.7
Neointima volume [mm^3^]	28.16 ± 15.10
Mean neointima burden [%]	24.6 ± 8.6
Neoatherosclerosis assessment	
Type I (thin cap, lipid-rich)	1 (3.4)
Type II (thick cap, layered)	4 (13.8)
Type III (peristrut, homogenous)	10 (34.5)
Type IV (preexisting, homogenous)	14 (48.3)

OCT analysis based on *n* = 29 cases (14.2 %)

The morphological analysis revealed that thin-cap atheroma was present only in 1 (3.4 %) case, and in the majority (82.8 %, *n* = 24) the neointima was homogenous with plaque presence only around the stent struts.

## Discussion

This is the first study presenting clinical outcomes of Prolim® stent deployment in all-comers with *de novo* coronary lesions with the use OCT for the precise assessment of vascular healing. The main findings of this study confirmed that Prolim® stent characterized the very good performance with MACE and clinically driven TLR rates at 12 months of 6.9 % and 5.4 %, respectively. Moreover, the long-term safety was confirmed by the lack of thrombosis cases and the extremely low number of uncovered stent struts as well as by the homogenous structure of neointima in the OCT analysis.

The deployment of a durable polymer DES (DP-DES) is a standard of care in patients with coronary artery lesions. However, studies assessing biodegradable polymer DES (BP-DES) proved the non-inferiority to DP-DES with the expectation for the decreased inflammatory response after stent implantation and, as a consequence, for faster vessel healing [12]. Moreover, sirolimus-eluting BP-DES and DP-DES showed similar results in STEMI patients [13]. The Prolim® study group consisted of patients with both stable coronary artery disease as well as acute coronary syndromes, including STEMI and NSTEMI. Moreover, 3.9 % of stented lesions were located in saphenous grafts, 38.8 % - within bifurcation lesions and 36.7 % - in complex lesions (type B2/C). The primary study endpoint occurred in 6.9 % of patients (*n* = 14), and clinically-driven TLR rate was 5.4 % (*n* = 11). There was no death or in-stent thrombosis.

These results are in line with previous studies with BP-SES implantation. In the study assessing Supralimus® stent (Sahajanand Medical Technologies, Gujarat, India) MACE rate was 0 % after one month, 6 % at 9-month follow-up and 7 % after 30 months follow-up [14].

Initial results of the prospective trial assessing Excel® stent (BP-SES) showed that at 12 months MACE and TLR rates were both 4 % [15]. Whereas in the BIOFLOW III Registry with Orsiro® (Biotronik, Berlin, Germany) stent the TLR rate was 5.1 % [16]. Worth mentioning is the BIOFLOW II trial, in which Orsiro® stent was compared with everolimus-eluting Xience® stent (MACE at 1 year SES 6.5 % versus EES 8.0 %) [17]. Moreover, in the ISAR-TEST-4 study patients presenting with stable coronary disease or acute coronary syndromes undergoing DES implantation in de novo coronary lesions were randomly assigned to treatment with BP-DES (sirolimus-eluting; *n* = 1299) or DP-DES (*n* = 1304: sirolimus-eluting, Cypher® or everolimus-eluting, Xience®). BP-DES was non-inferior to DP-DES concerning the primary endpoint (13.8 % vs. 14.4 %, *p* = 0.66) and showed similar rates of cardiac death or MI related to the target vessel (6.3 % vs. 6.2 %, *p* = 0.94), TLR (8.8 % vs. 9.4 %, *p* = 0.58), and stent thrombosis (definite/probable: 1.0 % vs. 1.5 %, *p* = 0.29) [18].

At 12 months the late lumen loss of the Prolim® stent was 0.21 ± 0.18 mm. Worth mentioning is ISAR-TEST3 study, in which patients with *de novo* coronary lesions were randomly assigned to receive a BP-DES, a polymer-free DES stent or DP-DES (Cypher®). The mean late lumen loss at 6–8-month follow-up was 0.17 ± 0.45 mm in the BP stent group, 0.23 ± 0.46 mm in the Cypher® cohort, and 0.47 ± 0.56 mm in the polymer-free stent group [19]. In previously mentioned studies the late lumen loss values were as followed: for Excel® stent - 0.12 ± 0.34 mm, for Orsiro® stent – 0.10 ± 0.32 mm, for Xience® stent – 0.11 ± 0.29 mm, and for Supralimus® stent – 0.09 ± 0.37 mm [14, 15, 17]. Simultaneously one must underline that the Prolim® stent is a relatively thick-strut DES, which platform is made of stainless steel 316 L (115 μm). And as earlier studies showed this might be associated with worse clinical and angiographic outcomes both in BMS as well as in DES [20–22]. However, in our study higher LLL value did not translate into worse clinical outcomes.

The safety profile of the stent is confirmed by a high rate of the device success (99 %) and a low rate of periprocedural complications. The rate of MI type 4a of 3.4 % might have been associated with the fact that certain implantations were performed in lesions located within bifurcations or venous grafts. Worth mentioning is the fact that despite the relative small stent cells size (for Prolim® stent of 3 mm in diameter, the maximal diameter of the expanded stent cell is 1.8 mm), the side branch occlusion was not nearly (0.98 %) observed. The second issue associated with safety is the rate of thrombosis. In case of Prolim® stent there was no early or late in-stent thrombosis. Presently, stent thrombosis in second or third generation stents is a rare clinical scenario [23]. Also, as confirmed in several meta-analyses BP-DES compared with DP-DES are associated with a lower rate of very late stent thrombosis and an equivalent risk of MACE [24, 25]. This probably might be associated with the fact that over time, the drug-coated polymers of BP-DES can gradually degrade into completely harmless CO_2_ and H_2_O molecules that are excreted from a patient’s body. In this manner, a BP- DES can completely transform into a BMS following a slow, controlled drug release. Thus, comparing to DP-DES, BP-DES can reduce drug-induced delays in vascular endothelialization and inflammatory responses of local vessel walls caused by the presence of a permanent polymer, thereby achieving the dual purpose of preventing both in-stent restenosis and late stent thrombosis [26].

Safety is always the major concern in a novel DES. Initial OCT studies reported that BP-SES characterized faster neointima coverage comparing with durable polymer SES [27]. Therefore, we performed an OCT subgroup analysis to better understand the endothelialization process over time. We found almost complete vessel healing with 99.3 % stent strut coverage 12 months after the index procedure. This was superior to the 96.5 % coverage rate of everolimus-eluting stent Xience V® (Abbott Vascular, Santa Clara, CA) and the 93.5 % coverage rate of the zotarolimus-eluting stent Resolute Integrity® (Medtronic Inc., Santa Rosa, CA) in the 13-month OCT substudy of the RESOLUTE All Comers trial [28]. Also, this was in line with an OCT substudy of LEADERS trial, in which BP-DES characterized a more complete stent coverage (99.4 %) as compared with DP-DES (97.9 %) at 9 months follow-up [29]. Also, the RUTTS score (Ratio of Uncovered to Total Stent Struts Per Cross Section) indicated very good healing (RUTTS ≤ 30 % = 100 %). This is crucial since RUTTS score is a useful predictor for late stent thrombosis [30]. Moreover, no thrombi were detected under OCT inspection. However, this excellent strut tissue coverage might have been achieved at the price of excessive neointimal growth. In OCT analysis mean neointima volume was 28.16 ± 15.10 mm^3^ and mean neointima burden was 24.6 ± 8.6 %. These findings correlated with mean neointimal area per section of 2.17 ± 1.37 mm^2^ that was thicker as compared with that observed in BP-DES in paper by Davlouros (0.4 mm^2^) or in NOBORI stent (biodegradable polymer, biolimus-eluting stent, 0.5 mm^2^) [31, 32]. However, recently published STACCATO trail showed in OCT a significantly higher percentage of uncovered struts in the BioMatrix BES stent compared with the XIENCE EES at nine-month follow-up. This study presented the opposite findings and did not support a preferential use of stents with biodegradable polymer-based biolimus elution to reduce the risk for stent thrombosis [33].

Ultimately, OCT results provided the additional insight in the characteristics of neointima formation in BP-DES at 12 months after its implantation. In most cases homogenous peristrut or preexisting atheroma (82.8 %, *n* = 24) was observed, and only in 4 (13.8 %) cases there was layered neoatheroslerosis, and in 1 (3.4 %) case there was a heterogenous, thin-cap neoatheroma. These data are crucial since the homogeneous neointima pattern correlated in earlier reports with a high proportion of connective tissue and smooth muscle cells in histopathology indicating favorable vessel healing, whereas, heterogenous neointima was found to correlate with higher presence of fibrin as compared to homogenous one and was associated with poorer clinical outcomes. Also the layered neointima corresponded with peristrut inflammation, and a small proportion of layered neointima suggested a lower inflammatory response to BP-DES as compared with DP-DES [34, 35]. However, one must remember that OCT imaging is not as good as fractional flow reserve measurements in predicting the significance of the coronary lesion [36].

Clinical results of our study have proved the safe profile of healing process after Prolim® stent implantation and indirectly have confirmed that similarly to other trials with BP-DES this particular stent does not require a prolonged dual antiplatelet therapy since it increases the risk of bleeding, and might be associated with adverse cardiac events at 1-year follow-up [37].

## Study limitations

This registry has several limitations that should be acknowledged. First of all the sample size was relatively small and no sample size calculation was performed. Other limitations of this study are its non-randomized manner and all known drawbacks of registry studies. The study was based mainly on clinical follow-up and control coronary angiography was performed in half of patients therefore some Prolim® stent failures might have been missed. However, despite this it was not restricted only to symptomatic patients and in addition a representative part was supplemented with OCT analysis.

## Conclusions

Sirolimus-eluting Prolim® stent with a biodegradable polymer is a feasible device with a very good safety profile and long-term clinical effectiveness. The OCT analysis showed excellent vessel healing with homogenous neointima proliferation. (ClinicalTrials.gov identifier: NCT02545985)

## Abbreviations

ACS: Acute coronary syndrome
CK: Creatine kinase
CK-MB: Ceatine kinase myocardial band
BP-DES: Biodegradable polymer drug eluting stent
DES: Drug eluting stent
DP-DES: Durable polimer drug eluting stent
LLL: Late lumen loss
MACE: Major cardiovascular adverse events
MI: Myocardial infarction
NSTEMI: non-ST elevation myocardial infarction
OCT: Optical coherence tomography
QCA: Quantitative coronary angiography
PCI: Percutaneous coronary intervention
SCAD: Stable coronary artery disease
STEMI: Myocardial infarction
TLR: Target lesion revascularization
TVR: Target vessel revascularization
TnI: Troponin I
UA: Unstable angina
